# Supplementary material for: Linear interaction between replication and transcription shapes DNA break dynamics at recurrent DNA break Clusters
Source: Nat Commun. 2024 Apr 27;15:3594. doi: 10.1038/s41467-024-47934-w (PMC11055891; doi:10.1038/s41467-024-47934-w)
Supplement: Supplementary file 1 — Supplementary Information [file 41467_2024_47934_MOESM1_ESM.pdf]

Linear Interaction Between Replication and Transcription Shapes DNA Break Dynamics at Recurrent DNA Break Clusters

Lorenzo Corazzi[1,2,#], Vivien Ionasz[1,2,#], Sergej Andrejev[1], Li-Chin Wang[1], Athanasios Vouzas[3,4], Marco Giaisi[1], Giulia Di Muzio[1,2,5], Boyu Ding[1,2,6], Anna J.M. Marx[1,2,5], Jonas Henkenjohann[1,2,5], Michael M. Allers[1,6], David M. Gilbert [4], and Pei-Chi Wei[1,2,5*]

Affiliations

1. German Cancer Research Center, 69120 Heidelberg, Germany.
2. Faculty of Bioscience, Ruprecht-Karl-University of Heidelberg, 69120 Heidelberg, Germany.
3. Department of Biological Science, Florida State University, FL 32306, United States of America.
4. San Diego Biomedical Research Institute, San Diego, CA 92121, United States of America.
5. Interdisciplinary Center for Neurosciences, Ruprecht-Karl-University of Heidelberg, 69120 Heidelberg, Germany.
6. Faculty of Medicine, Ruprecht-Karl-University of Heidelberg, 69120 Heidelberg, Germany.

\# These authors contributed equally.

*. To whom correspondence should be addressed: p.wei@dkfz-heidelberg.de.

SUPPLEMENTARY INFORMATION

SUPPLEMENTARY METHODS

High-resolution Repli-seq sample preparation

To facilitate reproducible sorting windows, the region from the G1 peak to the midpoint between the G2 peaks was uniformly sliced into 16, resulting in S1–S16 fractions. Cells to the left of the G1 peak were designated as the G1 fraction. For each S phase fraction, 80,000 cells were collected, and for the G1 fraction, 200,000 cells were gathered. After cell collection, total genomic DNA was extracted from cells in each fraction, and DNA was fragmented with a Covaris S220 Focused-ultrasonicator to 200-bp average fragment size. The DNA from S fractions S1–S16 was processed into libraries with a modification to the protocol outlined by Zhao et al. After shearing the genomic DNA and ligating adaptors (NEBNext® Ultra™ II DNA Library Prep Kit for Illumina, E7645L), the adaptor-ligated DNA from 80,000 cells was subjected to BrdU immunoprecipitation using 0.5 µg of anti-BrdU Santa Cruz sc-32323 (dilution: 1:200) for 30 minutes at room temperature. The BrdU-DNA/anti-BrdU complex was then captured by five µL of Dynabeads Protein G (Thermofisher #10003D) added directly to this reaction for 30 minutes at room temperature. Following capture, the BrdU-DNA/anti-BrdU/Protein G bead complexes were washed with 200 µL of PBST three times (5 minutes each) before the release of BrdU-DNA through Proteinase K digestion and purification, as

previously described by Zhao et al., prior to library indexing. We followed the NEBNext Multiplex Oligos for Illumina (New England BioLabs, E7645) protocol to introduce a dual index for the repli-seq libraries. Repli-Seq libraries were sequenced on NovaSeq 6000 to generate 100 bases of single-ended reads. Reads were aligned to mouse genome mm10 using Bowtie2. The alignment and coverage conversion pipeline is available through GitHub (https://github.com/brainbreaks/HighRes_RepliSeq).

LAM-HTGTS Libraries used in this article

For RDC detection, we combined samples from published data[1,2] (GSE106822 and GSE74356, 59 APH treated and 59 DMSO control) and newly generated samples from Chr5, 6, 8, 12, and 17 baits in Xrcc4-/-p53-/- ES cell-derived NPCs (GSE233842, 30 APH treated and 19 DMSO control). The new datasets (23 APH-treated and 19 untreated LAM-HTGTS libraries) generated from this article were sequenced under NextSeq 550. NextSeq produces five times more reads per library than its predecessor, Miseq, which was used to produce LAM-HTGTS libraries for the published datasets (59 APH-treated and 59 untreated LAM-HTGTS libraries by [1,2]). APH-treated inter-chromosomal and intra-chromosomal DSB were used for the final list of RDCs. Because we observe priming strand bias at Dtel and Dcen on the bait chromosomes, analyses that involve separating LAM-HTGTS DSB directionalities are performed using inter-chromosomal DSB. The terminology “junction” and “DSB” are interchangeable in the context of LAM-HTGTS experiments.

LAM-HTGTS Data clean-up

To eliminate DSBs that originated from resection around the bait viewpoint bait DSB, junctions from the bait viewpoint region (±6Mb, 2690102 junctions) and bait off-target regions (±50Kb, 58931 junctions) were removed. Also, duplicate reads (176774 junctions) and reads with multiple alignments (292903 junctions) were removed. Additionally, intra-chromosomal junctions from libraries where the bait chromosome was genetically modified were excluded from the subset used for RDC calling (270332 junctions). The final dataset contained 1116629

reads distributed across four conditions: APH-Inter (517947), APH-Intra (152146), DMSO-Inter (365270), and DMSO-Intra (81266).

Offtarget calling

Junctions were extended 150bp in the opposite direction from translocated prey, and only junctions that overlapped with opposite-direction junctions were kept so that there was an equal amount of centromeric and telomeric-oriented junctions. A pileup was calculated from the resulting junctions, and a Poisson distribution (mean=2) was used to calculate the significance for each interval. Continuous significant regions (p-value<0.01) were further filtered to contain more telomeric translocations upstream from the off-target site and more centromeric translocations downstream (Fisher exact test, p-value<0.01).

DRIP-seq and DRIPc-seq library preparation

ES cell-derived NPCs at 75-80% confluency were detached by incubating with accutase at 37°C for eight minutes. Single-cell suspension was collected, and cells were pelleted down by centrifugation at 250 x g for three minutes at 4°C. Cells were washed in cold DPBS, pelleted down by centrifugation again, and resuspended in TE buffer. Ten million cells were used for the following steps. Cells were digested with proteinase K at 56°C overnight, and the genomic DNA was purified with phenol/chloroform extraction as described in the protocol. Genomic DNA was digested in a restriction enzyme cocktail (HindIII, EcoRI, XbaI, BsgRI, and SspI) to derive a DNA smear between 500bp – 2kb in size. Fragmented genomic DNA was extracted following a phenol/chloroform protocol, and 10 μg genomic DNA was used for each DRIP reaction. The genomic DNA was split into two reactions. Reaction one was treated with RNAseH to remove DNA:RNA hybrid in vitro. For RNAseH-treated controls, genomic DNA was incubated with 20 units RnaseH (M0297S, NEB) at 37°C for 4-6 hours. Reaction two did not receive RNAseH treatment, thus preserving the DNA:RNA hybrid structure. DNA was purified from both reactions for DNA:RNA immunoprecipitation (DRIP). For each DRIP, 20 μg S9.6 monoclonal antibody (affinity purified from the S9.6 hybridoma

culture medium; 1:25) was used for DNA::RNA pulldown. Antibody-DNA::RNA complex was then pulldown with 50 μL protein G Dynabeads (10003D, Thermal Fisher Scientific). DNA::RNA complex was eluted, and the enrichment was validated by quantitative PCR at the known R-loop loci (beta-Actin, EIF5alpha) and the non-R-loop loci (chr1). Primer sequences were described in previously published protocol[3]. For DRIPc-seq, 30 units of DNAse I (New England BioLabs, M0303S) was added to the DNA::RNA complex to degrade DNA. RNA was precipitated in 75% ethanol/0.3M sodium acetate, washed in 75% ethanol, and dissolved in 10 mM Tris-Cl, pH 8. The RNA molecules were reverse transcribed (IScript reverse transcription supermix, Bio-Rad, 1708840), and the second strand was synthesized using the cocktail described before [3]. RNA present in the prep were degraded with 5U RNase H (New England BioLabs; M0297S). DRIPc-seq libraries were constructed as described for DRIP-seq.

DRIP- and DRIPc-seq data analyses

Libraries were performed as described and were sequenced on Illumina Nextseq (75 bp single end). After sequencing, the adaptors were trimmed from the raw FASTQ reads, and the reads were aligned to mm10/GRCm38 through Bowtie2. The SAM files were transformed to BAM, sorted, and subject to MACS2 peak calling. We used the default MACS2 function, treating two DRIP samples as “treatment” and one input sample as “control.” Peaks smaller than 250 bp, with fold induction less than 10, and p or q value greater than 10^{-10} were eliminated from downstream analyses. To determine the strandness of DNA:RNA hybrids, the enrichment was called using BAM files containing either plus or minus DRIPc-seq reads with the same MACS2 parameter described above. We then applied the bedtools intersect function to identify genomic regions that contain both plus and minus strand peaks. These peaks are defined as the dual-strand DNA:RNA hybrids. We used the bedtools intersect function to extract DRIPc-seq peaks at the coding orientation in genes annotated at mm10 Refgene. DRIP-seq and DRIPc-seq enrichment (Supplementary Figure 5) were calculated using computeMatrix and plotted via the plotHeatmap function in deeptools.

SUPPLEMENTARY FIGURES

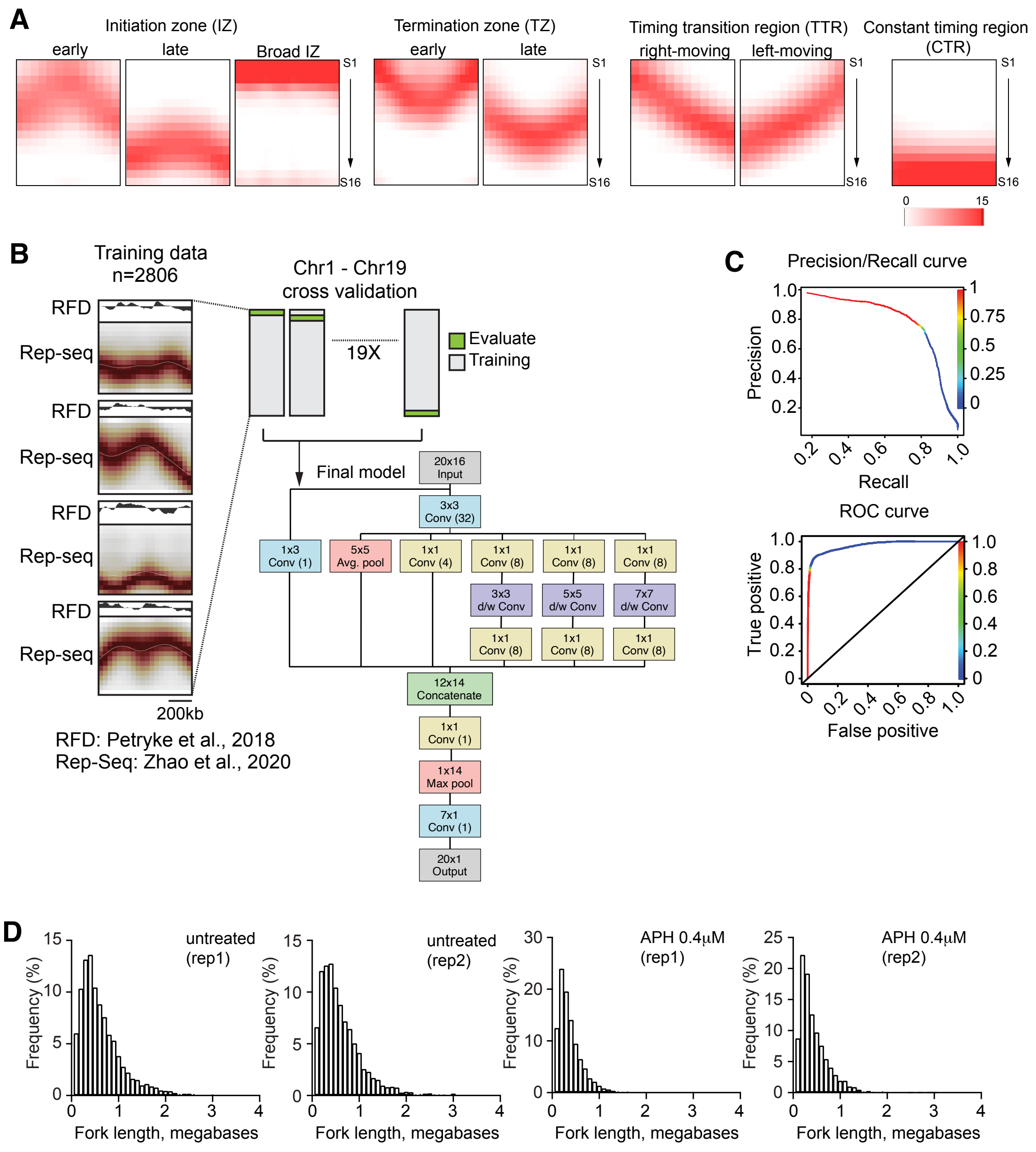

Supplementary Figure 1. Replication Features Revealed by High-Resolution Repli-seq. Related to Figure 1. (A) Representation of annotated features found in the high-resolution Repli-seq normalized heatmaps. **(B)** The convolutional neural network and the determination of replication directions. The diagram presents a convolutional neural network to identify replication termination zones based on OK-seq and Repli-seq data. Further information on selecting training regions can be found in the Methods section. On the left, four example training regions display Replication Fork Directionality (RFD) signals obtained from OK-seq

and the signal from 16-fraction Repli-seq data. The training process was conducted sequentially, employing a leave-one-out cross-validation strategy. Chromosome X was excluded from the training. **(C)** The figure shows the precision and performance results of convolutional neural network training. A recall and precision curve and a receiver operating characteristic (ROC) curve are shown. **(D)** Histograms display the distribution of lengths for replication forks in two untreated and two 0.4 μM APH-treated ES cell-derived NPCs samples as determined by the convolutional neural network. 2190 and 2350 meeting points in the untreated and 3068 and 3275 meeting points in the aphidicolin-treated XRCC4/p53-deficient NPC were plotted.

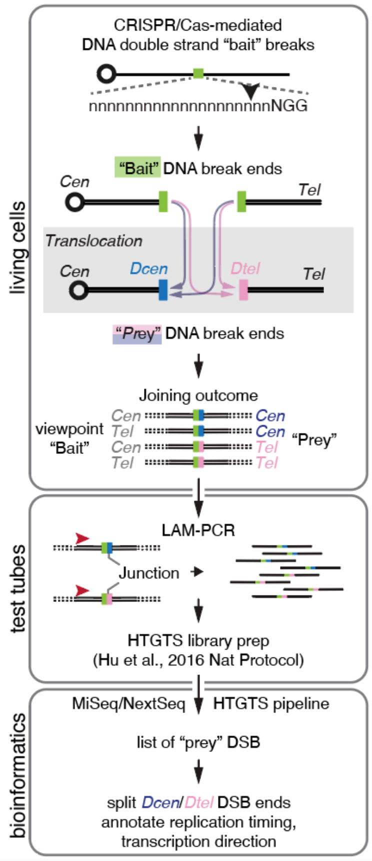

Supplementary Figure 2. Using LAM-HTGTS to determine DSB end orientation. Related to the main text and Figure 2. The LAM-HTGTS workflow and differentiation of centromeric and telomeric DSB ends. A bait DSB is introduced through the CRISPR/Cas system within living cells. The position of the DSB is indicated by a black vertical arrow located at the sgRNA targeting sequences. Upon CRISPR/Cas9 cutting, it generates two DSB ends to be used as bait viewpoints. These ends are highlighted as green rectangles. "Cen" and "Tel" correspond to centromere and telomere. These centromeric and telomeric DSB ends interact at the bait viewpoint and connect with "prey" DSBs on the same or different chromosomes. This process leads to an equivalent frequency of translocations from the bait DSB to either the centromeric or telomeric prey DSB ends when both are available. The translocation involving prey DSBs pointing in the telomeric direction is denoted as "Dcen " or "Dtel," based on the orientation. Genomic DNA is subsequently extracted for further procedures. The genomic DNA was fragmented, and a single primer (indicated by a red arrowhead) positioned upstream of the bait viewpoint DSB is employed for linear-amplification mediated PCR (LAM-PCR). This procedure maintains the strand orientation of prey sequences and DSB orientation. The

subsequent steps of LAM-HTGTS library preparation adhere to the protocol established before [4]. In our approach, we differentiate DSBs with a "Dcen " tag from those with a "Dtel" tag, as these represent DSBs with open ends associated with centromeric sequences or telomeric sequences, respectively.

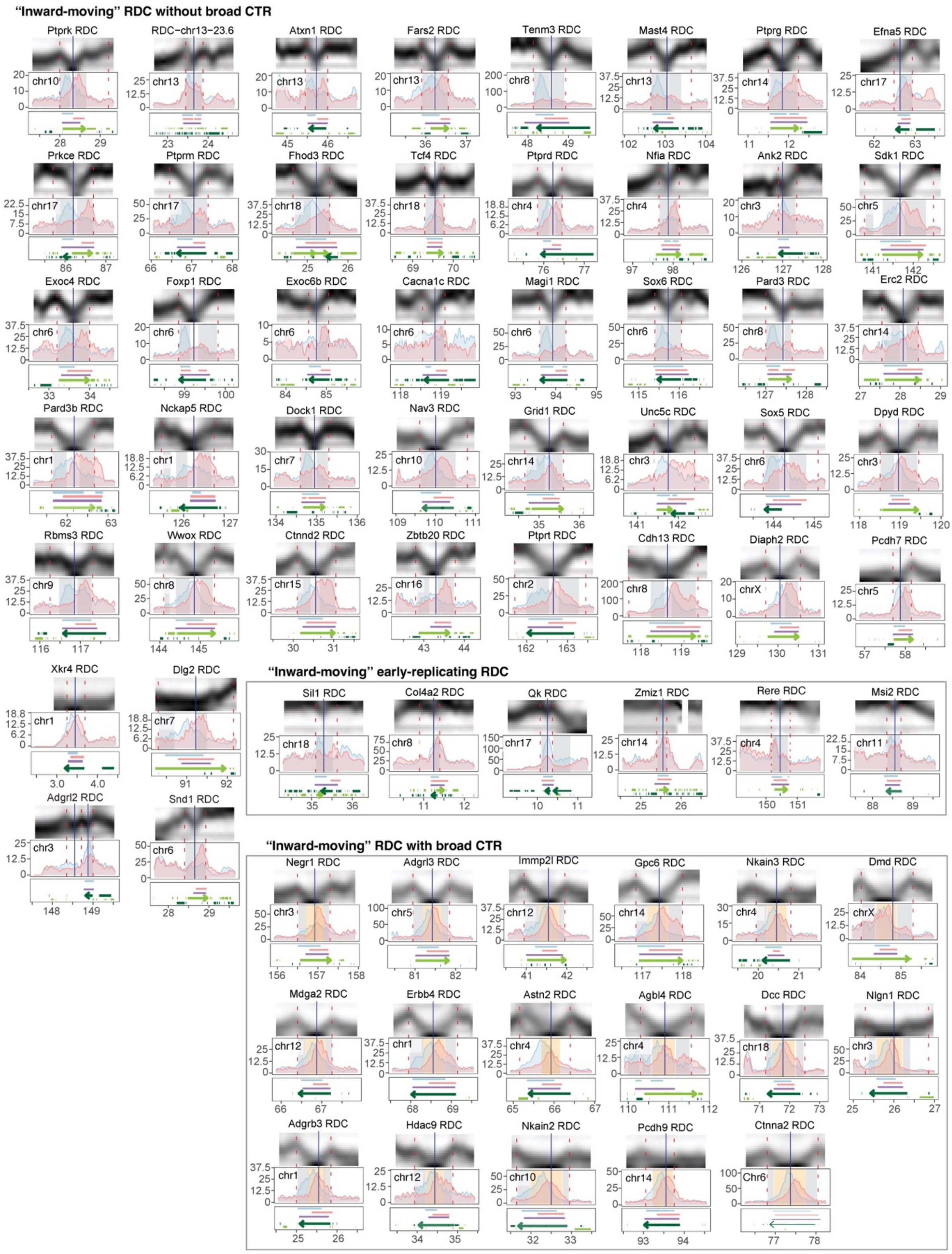

Supplementary Figure 3. Multi-omics Data Showing Information on Genomic Sequences around "Inward-Moving" RDC. Related to Figure 2. Panels are presented as described in Figure 2C, except that we omitted annotating replication fork directions due to the complexity of plots. Timing transition region (TTR) were shaded in gray, and late constant timing region greater than 500 kb were shaded in yellow.

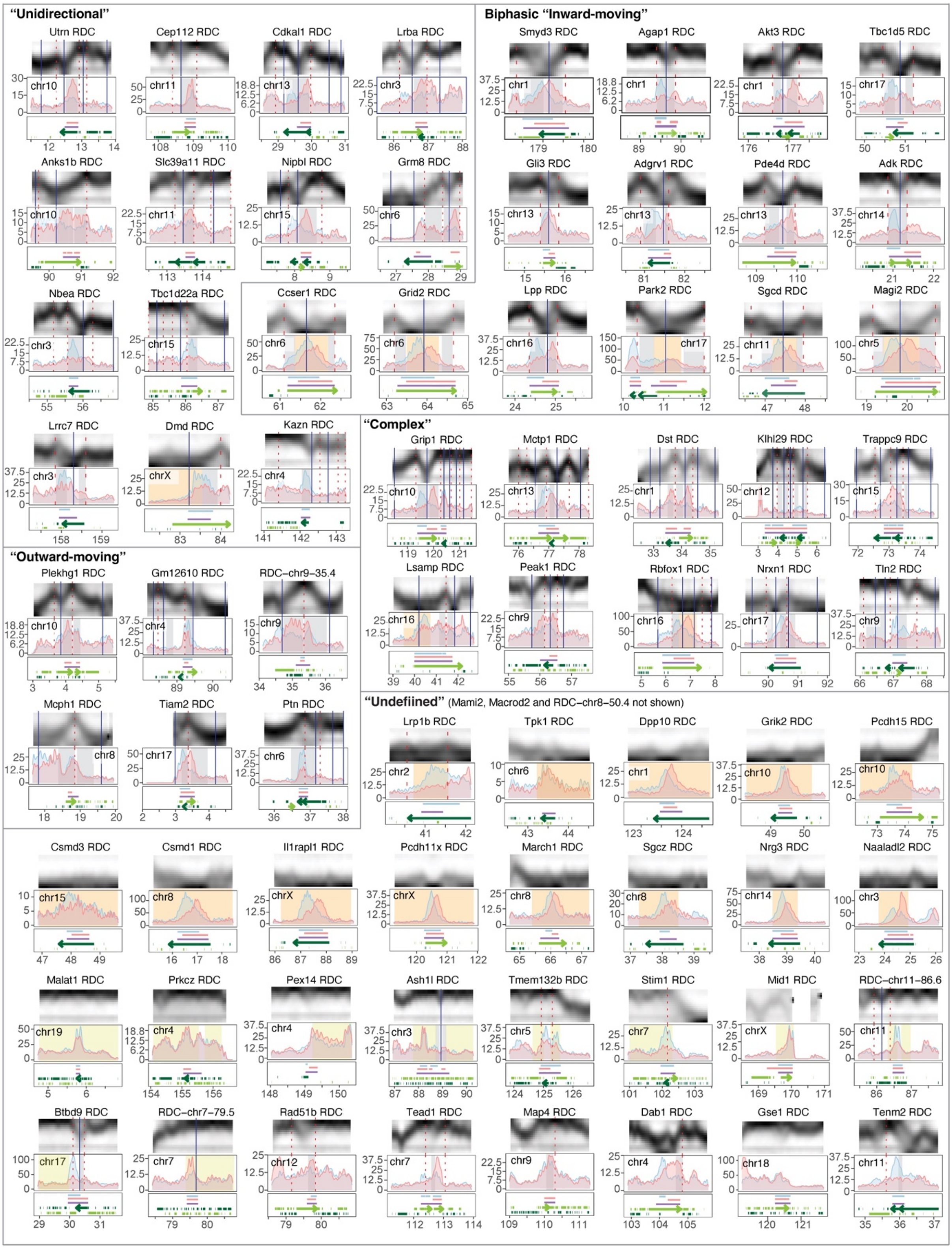

Supplementary Figure 4. Multi-omics Data Showing Information on Genomic Sequences around "Unidirectional," "Complex," Biphasic, "Inward-Moving," "Outward-Moving," and "Undefined" RDC. Related to Figure 3 and main text. Panels are presented as described in Figure 2C without annotating replication fork directions. The broad initiation zone

was shown in light green rectangular. Timing transition region (TTR) were shaded in gray, and late constant timing region greater than 500 kb were shaded in yellow. Broad initiation zones were shaded in grass green.

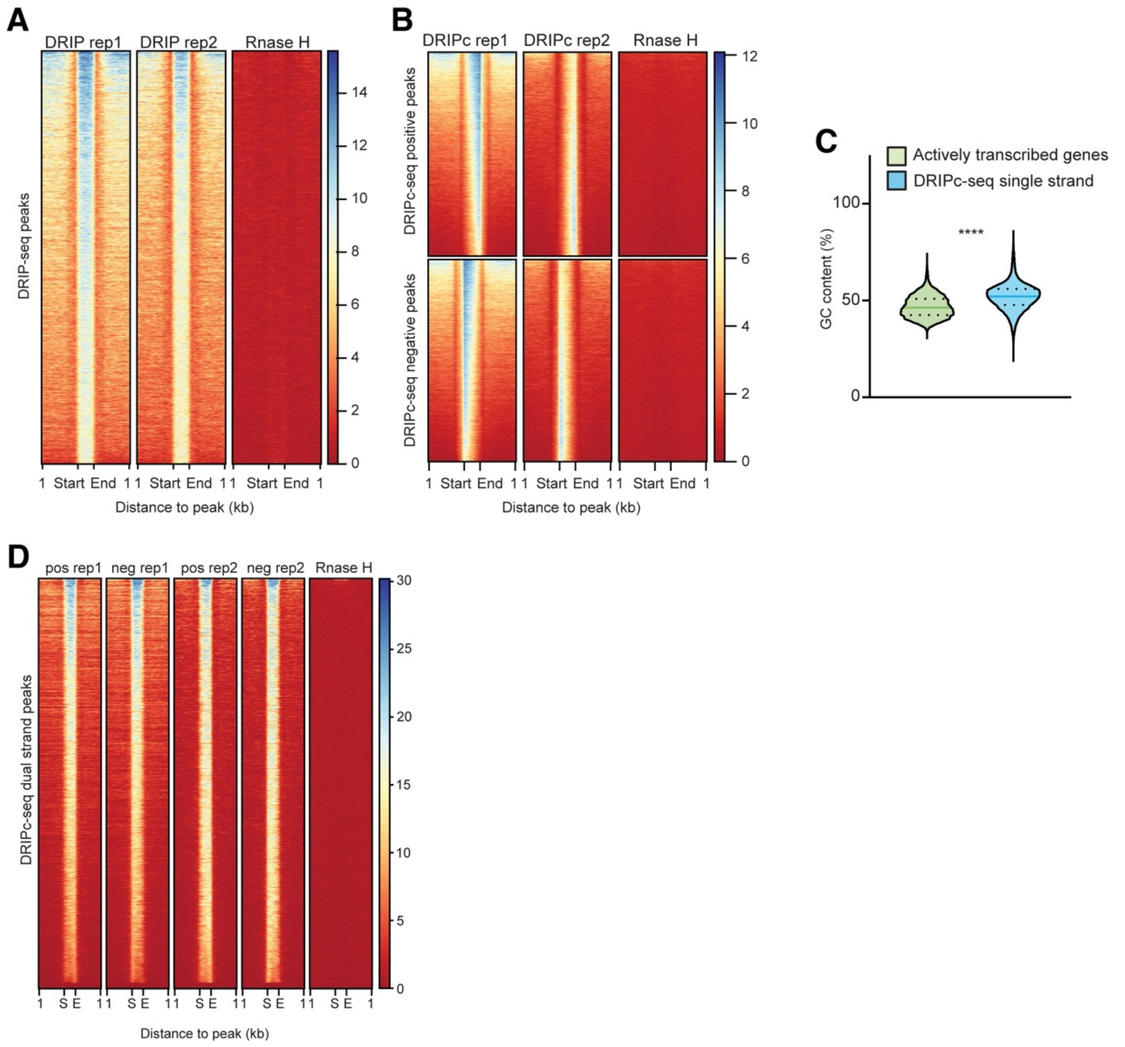

Supplementary Figure 5. DRIP-seq and DRIPc-seq Related information. Related to Figure 5. (A, B, D) The scores per genomic region at and around the 8,822 DRIP-seq peaks sensitive to RNase H treatment **(A)**, 33,933 RNase H-sensitive, coding strand-specific DRIPc-seq peaks **(B)**, and 11,386 dual-strand DRIPc-seq peaks **(D)** are shown as heatmaps. The corresponding heatmap scores are colored as indicated in a vertical bar to the right of each figure. The start and end denote the DRIPc-seq peak region. **(C)** GC content of genomic sequences underneath the DRIPc-seq stranded peaks (n=42,049) and underneath actively transcribed genes (n=14436). The two-tailed Mann-Whitney test determined the statistical power: ****P<0.0001.

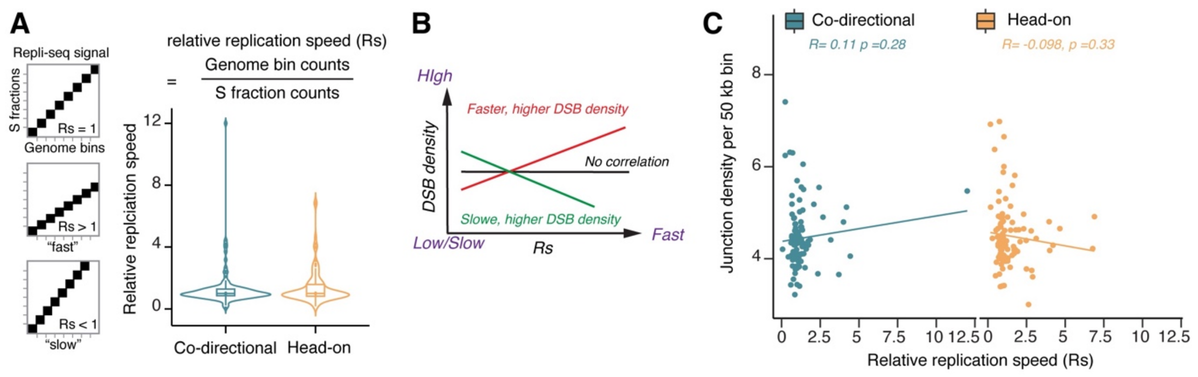

Supplementary Figure 6. DNA Break Density at the Head-on and Co-directional TRC at RDC. Related to Figure 6. (A) Left: Illustrations portray the approach employed for calculating the relative replication speed. A schematic depicts an 8 x 8 50-kb bin square representing the replication of 400 kb genomic sequences across eight S fractions. The Repli-seq signal is denoted by black squares, illustrating three scenarios of replication speed. On the right: Violin plots display the distribution of relative replication speed (Rs) for co-directional and head-on TRCs at the "inward-moving," "unidirectional," and "complex" RDCs. 107 co-directional and 101 head-on TRC were analyzed. The formula utilized to compute Rs is presented on top. The boxes within the violin plots denote the 25 – 75% quartiles, with the median indicated by a horizontal line. **(B)** A scheme depicting the correlation between DNA break density and Rs. The colored lines represent fitted exponential regression outcomes. **(C)** Scatter plots showing the DNA break density and Rs at "inward-moving", "unidirectional", and "complex" RDC within the codirectional (left) or the head-on TRC (right). The average size for co-directional TRC is 331 kb, while head-on TRC is 351 kb. The relationship between DNA break density and Rs was fitted with exponential regression lines. Y axis: interchromosomal Dcen or Dtel density per 50 kilobases bin. X-axis: relative replication speed. The R-squared and P-values of the regression line were indicated below the scatter plots.

SUPPLEMENTARY REFERENCES

1. Wei, P.-C. *et al.* Long Neural Genes Harbor Recurrent DNA Break Clusters in Neural Stem/Progenitor Cells. *Cell* **164**, 644–55 (2016).

2. Wei, P.-C. *et al.* Three classes of recurrent DNA break clusters in brain progenitors identified by 3D proximity-based break joining assay. *Proc National Acad Sci* **115**, 1919–1924 (2018).

3. Sanz, L. A. & Chédin, F. High-resolution, strand-specific R-loop mapping via S9.6-based DNA-RNA immunoprecipitation and high-throughput sequencing. *Nat Protoc* **14**, 1734–1755 (2019).

4. Hu, J. *et al.* Detecting DNA double-stranded breaks in mammalian genomes by linear amplification–mediated high-throughput genome-wide translocation sequencing. *Nat Protoc* **11**, 853–871 (2016).
