# Supplementary material for: Linear interaction between replication and transcription shapes DNA break dynamics at recurrent DNA break Clusters
Source: Nat Commun. 2024 Apr 27;15:3594. doi: 10.1038/s41467-024-47934-w (PMC11055891; doi:10.1038/s41467-024-47934-w)
Supplement: Supplementary file 3 — Description of Additional Supplementary Files [file 41467_2024_47934_MOESM3_ESM.pdf]

Description of Additional Supplementary Files

File Name: Supplementary Data 1
Description: Assay types are used in this article, as well as data and analytical pipeline availability.

File Name: Supplementary Data 2
Description: The Excel spreadsheet encompasses information about the 152 RDCs analyzed in this study, including their respective chromosome (RDCChrom), start (RDCstart), and end (RDCend) coordinates, the associated gene (> 100kb) that colocalizes with each RDC, the corresponding RDC name (Gene (RDC) name), the coding strand of the gene (Gene Coding strand), the start (Gene start) and end (Gene end) coordinates of the indicated genes, the negative logarithm of the P value of the RDC (-log10P), if an RDC was described previously, and the RDC group classification. The P value was determined by the Goodness-of-fit tests using the gamma distribution by maximum likelihood estimation followed by Benjamini-Hochberg correction.

File Name: Supplementary Data 3
Description: Gene Ontology (GO) analyses for previously and newly identified RDC-containing genes, as well as s containing TTR in NPCs. The statistic power was determined by a multi testing approach with the family-wise error rate estimation. -logP value smaller than 3 were reporter in the list. The P value was determined by a two-sided hypergeometric test and corrected by family-wise error rates. This analysis was done by using GofuncR: Gene ontology enrichment using FUNC. R package version 1.22.2.

File Name: Supplementary Data 4
Description: Coordinates for replication features characterized in aphidicolin-treated NPCs.

File Name: Supplementary Data 5
Description: The extension for Supplementary Table 2. The Excel spreadsheet encompasses information about (1) junction numbers at Dcen or Dtel direction, (2) the shape of RDC DSB peaks, (3) replication direction, (4) biphasic status, (5) replication features, and (6) number of DRIP- and DRIPc-seq peaks in each RDC.

File Name: Supplementary Data 6
Description: The Excel spreadsheet comprises oligo sequences and reagent details employed in this research. The specific function of each oligo and reagent is indicated.
