# Supplementary material for: Linear interaction between replication and transcription shapes DNA break dynamics at recurrent DNA break Clusters
Source: Nat Commun. 2024 Apr 27;15:3594. doi: 10.1038/s41467-024-47934-w (PMC11055891; doi:10.1038/s41467-024-47934-w)
Supplement: Supplementary file 10 — Reporting Summary [file 41467_2024_47934_MOESM10_ESM.pdf]

nature portfolio

Corresponding author(s): Pei-Chi Wei

Last updated by author(s): Mar 20, 2024

Reporting Summary

Nature Portfolio wishes to improve the reproducibility of the work that we publish. This form provides structure for consistency and transparency in reporting. For further information on Nature Portfolio policies, see our Editorial Policies and the Editorial Policy Checklist.

Statistics

For all statistical analyses, confirm that the following items are present in the figure legend, table legend, main text, or Methods section.

n/a	Confirmed	
☐	☒	The exact sample size (*n*) for each experimental group/condition, given as a discrete number and unit of measurement
☐	☒	A statement on whether measurements were taken from distinct samples or whether the same sample was measured repeatedly
☐	☒	The statistical test(s) used AND whether they are one- or two-sided<br>*Only common tests should be described solely by name; describe more complex techniques in the Methods section.*
☒	☐	A description of all covariates tested
☒	☐	A description of any assumptions or corrections, such as tests of normality and adjustment for multiple comparisons
☐	☒	A full description of the statistical parameters including central tendency (e.g. means) or other basic estimates (e.g. regression coefficient) AND variation (e.g. standard deviation) or associated estimates of uncertainty (e.g. confidence intervals)
☐	☒	For null hypothesis testing, the test statistic (e.g. *F*, *t*, *r*) with confidence intervals, effect sizes, degrees of freedom and *P* value noted<br>*Give P values as exact values whenever suitable.*
☐	☒	For Bayesian analysis, information on the choice of priors and Markov chain Monte Carlo settings
☒	☐	For hierarchical and complex designs, identification of the appropriate level for tests and full reporting of outcomes
☒	☐	Estimates of effect sizes (e.g. Cohen's *d*, Pearson's *r*), indicating how they were calculated

Our web collection on statistics for biologists contains articles on many of the points above.

Software and code

Policy information about availability of computer code

Data collection	Not applicable.
Data analysis	Codes and bioinformatic analysis pipelines were available at BrainBreaks GitHub (https://github.com/orgs/brainbreaks/repositories). Specifically, the data were analyzed by using the following packages: RDC calling and fork direction prediction (DOI: 10.5281/zenodo.10832658), LAM-HTGTS pipeline (DOI:10.5281/zenodo.10843397), GRO-seq (DOI: 10.5281/zenodo.10838367), and high resolution Repli-seq (DOI: 10.5281/zenodo.10838365). The link to package used for each assay is summarized in Supplementary Data 1.

For manuscripts utilizing custom algorithms or software that are central to the research but not yet described in published literature, software must be made available to editors and reviewers. We strongly encourage code deposition in a community repository (e.g. GitHub). See the Nature Portfolio guidelines for submitting code & software for further information.

Data

Policy information about availability of data

All manuscripts must include a data availability statement. This statement should provide the following information, where applicable:

- Accession codes, unique identifiers, or web links for publicly available datasets
- A description of any restrictions on data availability
- For clinical datasets or third party data, please ensure that the statement adheres to our policy

The raw and processed LAM-HTGT and GRO-seq data generated in this study have been deposited in the GEO database under accession code GSE233842 [https://

www.ncbi.nlm.nih.gov/geo/query/acc.cgi?acc=GSE233842]. The raw and processed Repli-seq, DRIP-seq and DRIPc-seq data are available under accession code GSE254765 [https://www.ncbi.nlm.nih.gov/geo/query/acc.cgi?acc=GSE254765]. The corresponding location for data generated in this study are provided in the Supplementary Data 1. In addition, the published LAM-HTGTS data used in this study are available in the GEO database under accession codes GSE106822 [https://www.ncbi.nlm.nih.gov/geo/query/acc.cgi?acc=GSE106822] and GSE74356 [https://www.ncbi.nlm.nih.gov/geo/query/acc.cgi?acc=GSE74356]. The sequencing data are available in the GEO database under accession code GSM3290342 [https://www.ncbi.nlm.nih.gov/geo/query/acc.cgi?acc=GSM3290342], and the CAST/F121-9 Repli-seq data used for convolution network training are available in the GEO database under accession code GSM137764 [https://www.ncbi.nlm.nih.gov/geo/query/acc.cgi?acc=GSM137764]. All sequences were mapped to the mouse genome mm10 (https://hgdownload.soe.ucsc.edu/goldenPath/mm10/bigZips/).

Research involving human participants, their data, or biological material

Policy information about studies with human participants or human data. See also policy information about sex, gender (identity/presentation), and sexual orientation and race, ethnicity and racism.

Reporting on sex and gender	Not applicable.
Reporting on race, ethnicity, or other socially relevant groupings	Not applicable.
Population characteristics	Not applicable.
Recruitment	Not applicable.
Ethics oversight	Not applicable.

Note that full information on the approval of the study protocol must also be provided in the manuscript.

Field-specific reporting

Please select the one below that is the best fit for your research. If you are not sure, read the appropriate sections before making your selection.

☒ Life sciences ☐ Behavioural & social sciences ☐ Ecological, evolutionary & environmental sciences

For a reference copy of the document with all sections, see nature.com/documents/nr-reporting-summary-flat.pdf

Life sciences study design

All studies must disclose on these points even when the disclosure is negative.

Sample size	For calling recurrent DNA break clusters (RDC), we performed a saturation test. 52 APH-treated samples are sufficient to discover 95% RDC and 26 samples revealed 80% RDCs. For this study, we included 82 APH-treated samples.
Data exclusions	LAM-HTGTS: Only DSB detected at the non-viewpoint chromosome are subjected to statistical analyses and plotting in Figures 2, 3, 4, 5, 6, and S3, 4, 5, 6. We excluded viewpoint-chromosome for analyses as the Dcen and Dtel recovery rate is unbalanced. The bait preferentially recovers 15-25% more downstream DSBs at the break site chromosome than the upstream. Using viewpoint chromosome DSB resulted in an overrepresentation of the centromeric DSB end when the bait had a centromeric orientation. The bait with a telomeric direction resulted in an overrepresentation of the telomeric DSB end. The bias due to bait DSB end orientation on the viewpoint chromosome was as large as 20%. DSB end recovery bias was not present on the non-viewpoint chromosome.
Replication	Data is derived from at least 3 technical repeats, all repeats were successful and where possible data used to perform statistical analysis.
Randomization	Depending on cell treatments as described, samples were assigned to corresponding groups in the manuscript.
Blinding	For peak calling, we extended LAM-HTGTS junctions by 50Kb symmetrically in both directions, and pileup islands were determined for telomeric-only (Dtel), centromeric-only (Dcen), and all junction orientations (Dtel + Dcen). A negative binomial model for estimating the expected pileup value for each chromosome/condition/junction-orientation triplet was derived, and a p-value was calculated for each pileup value concerning model expectation. This process is blinded from whether the junctions are enriched within gene or not. Regions with a p-value below 0.01 joined (maximal gap 10Kb) to create seeds. These seeds were further joined with other seeds (maximal gap 100Kb) to form islands. Islands are extended up and downstream to include regions below 0.1 significance. Overlapping orientation-specific islands are further joined to form an initial RDC list that is further filtered to contain at least 100Kb below 0.01 p-value and be of at least 300Kb in length when considering extended regions. The broadest range of all overlapping and significant islands determined RDC.

Reporting for specific materials, systems and methods

We require information from authors about some types of materials, experimental systems and methods used in many studies. Here, indicate whether each material, system or method listed is relevant to your study. If you are not sure if a list item applies to your research, read the appropriate section before selecting a response.

Materials & experimental systems

n/a	Involved in the study
☐	☒ Antibodies
☐	☒ Eukaryotic cell lines
☒	☐ Palaeontology and archaeology
☒	☐ Animals and other organisms
☒	☐ Clinical data
☒	☐ Dual use research of concern
☒	☐ Plants

Methods

n/a	Involved in the study
☐	☒ ChIP-seq
☒	☐ Flow cytometry
☒	☐ MRI-based neuroimaging

Antibodies

Antibodies used

αBr-dU antibodies, Santa Cruz#sc-32323, used for Repli-seq and GRO-seq.
S9.6 antibody, Millipore Sigma, cat. no. MABE1095, usef for DRIP-seq

Validation

BrdU (IIB5) is recommended for detection of BrdU, a proliferation marker incorporated into newly synthesized DNA during S-phase of a cell cycle, by immunofluorescence (starting dilution 1:50, dilution range 1:50-1:500), immunohistochemistry (including paraffin-embedded sections) (starting dilution 1:50, dilution range 1:50-1:500) and flow cytometry (1 µg per 1 x 106 cells); recognizes BrdU in denatured DNA of cells labeled with BrdU; may cross-react with iododeoxyuridine (IrdU). Anti-BrdU Antibody (IIB5) has 241 citations in a variety of scientific publications.

Clone S9.6 bound the DNA-RNA heteropolymer and poly(I)-poly(dC) equally, but 100-fold higher levels of poly(A)-poly(dT) were required to achieve a similar degree of binding. Single-stranded DNA, double-stranded DNA and RNA, and ribosomal RNA were not bound by clone S9.6 (Boguslawski, S.J., et al. (1986). J. Immunol Methods. 89(1):123-130). Target DNA-RNA heteroduplex (R loop) structure is not sequence- or species-specific. Evaluated by Immunocytochemistry in HeLa cells. Immunocytochemistry Analysis: A 1:50 dilution of this antibody immunolocalized nuclear and mitochondrial DNA-RNA hybrids in 4% paraformaldehyde-fixed, 0.3% Triton X-100-permeabilized HeLa cells. MABE1095 has 86 citations in a variety of scientific publications.

Eukaryotic cell lines

Policy information about cell lines and Sex and Gender in Research

Cell line source(s)

The original mouse Nxp010 Xrcc4/p53-deficient ES cells were described in Tena et al. (https://doi.org/10.1073/pnas.1922299117). This cell line has an X and a Y chromosome.

Authentication

For derivatives of the original cells generated during this study the genotype was verified by PCR amplification.

Mycoplasma contamination

All cell lines were tested for Mycoplasma using the EZ-PCR Mycoplasma testing kit (Biological Industries). Negative results were confirmed by including a +(ve) control provided by the kit.

Commonly misidentified lines
(See ICLAC register)

No commonly misidentified lines were used in this manuscript.

Plants

Seed stocks

Not applicable.

Novel plant genotypes

Not applicable.

Authentication

Not applicable.

ChIP-seq

Data deposition

☒ Confirm that both raw and final processed data have been deposited in a public database such as GEO.

☒ Confirm that you have deposited or provided access to graph files (e.g. BED files) for the called peaks.

Data access links
May remain private before publication.

Data can be found under GSE233842 and GSE254765. To access these private data, use tokens "gbsbuygutfcjbmn" for GSE233842 and "gjypiyeujpebnaj" for GSE254765.

Files in database submission

DRIPseq_NXP010_DMSO_S9.6_rep1_783897_bin5000-.bedgraph
DRIPseq_NXP010_DMSO_S9.6_rep2_783899_bin5000-.bedgraph
DRIPseq_NXP010_DMSO_S9.6_RNaseH1_rep1_783905_bin5000-.bedgraph
DRIPseq_NXP010_DMSO_S9.6_RNaseH1_rep2_783907_bin5000-.bedgraph
DRIPseq_NXP010_DMSO_input_rep1_783913_bin5000-.bedgraph

	High-resolution Repli-Seq bedGraph tracks, aphidicolin-treated, two replicates. Each replicate contains 16 S phase fractions.<br>Dripc-seq experiments, two replicates. Each replicate contains signals at the positive and negative strands.
Genome browser session<br>(e.g. UCSC)	https://genome.ucsc.edu/s/li-chin/mm10<br>https://genome.ucsc.edu/s/Peichiwei0804/NPC_highRes_RepliSeq_APH<br>https://genome.ucsc.edu/s/Peichiwei0804/NPC_dripc_seq

Methodology

Replicates	Two technical replicates were performed. All repeats were successful and where possible data used to perform statistical analysis.
Sequencing depth	DRIP-seq were performed on NextSeq550 with the Single-end 75 cycle chemistry. The total number of reads were between 35 - 38 million per ChIP condition. The reads number for input sample was 48 million. Overall, over 95% of the raw reads were uniquely mapped to mm10 genome.
Antibodies	S9.6 antibody, Millipore Sigma, cat. no. MABE1095, usef for DRIP-seq and DRIPc-seq
Peak calling parameters	We used the default MACS2 function, treating two DRIP samples as “treatment” and one input sample as “control.”
Data quality	Quality of the sequencing was assessed by FASTQC. Peaks smaller than 250 bp, with fold induction less than 10, and p or q value greater than 10^10 were eliminated from downstream analyses.
Software	MACS2 (https://doi.org/10.1186/gb-2008-9-9-r137)
